# Clustering analysis for the evolutionary relationships of SARS-CoV-2 strains

**DOI:** 10.1038/s41598-024-57001-5

**Published:** 2024-03-18

**Authors:** Xiangzhong Chen, Mingzhao Wang, Xinglin Liu, Wenjie Zhang, Huan Yan, Xiang Lan, Yandi Xu, Sanyi Tang, Juanying Xie

**Affiliations:** 1 School of Computer Science, Shaanxi Normal University, Xian, 710119 China; 2 College of Life Sciences, Shaanxi Normal University, Xian, 710119 China; 3 School of Mathematics and Statistics, Shaanxi Normal University, Xian, 710119 China

**Keywords:** Data mining, Health care, Machine learning

## Abstract

To explore the differences and relationships between the available SARS-CoV-2 strains and predict the potential evolutionary direction of these strains, we employ the hierarchical clustering analysis to investigate the evolutionary relationships between the SARS-CoV-2 strains utilizing the genomic sequences collected in China till January 7, 2023. We encode the sequences of the existing SARS-CoV-2 strains into numerical data through *k*-mer algorithm, then propose four methods to select the representative sample from each type of strains to comprise the dataset for clustering analysis. Three hierarchical clustering algorithms named Ward-Euclidean, Ward-Jaccard, and Average-Euclidean are introduced through combing the Euclidean and Jaccard distance with the Ward and Average linkage clustering algorithms embedded in the OriginPro software. Experimental results reveal that BF.28, BE.1.1.1, BA.5.3, and BA.5.6.4 strains exhibit distinct characteristics which are not observed in other types of SARS-CoV-2 strains, suggesting their being the majority potential sources which the future SARS-CoV-2 strains’ evolution from. Moreover, BA.2.75, CH.1.1, BA.2, BA.5.1.3, BF.7, and B.1.1.214 strains demonstrate enhanced abilities in terms of immune evasion, transmissibility, and pathogenicity. Hence, closely monitoring the evolutionary trends of these strains is crucial to mitigate their impact on public health and society as far as possible.

## Introduction

Neurological disorders^1^, diabetes^2^ and cognitive impairments^3^, particularly the pandemic COVID-19 in recent years have profoundly impacted human being. It is well known that this pandemic has been more than three years since the first confirmed case of COVID-19 caused by SARS-CoV-2 in 2019^4^, during which various strains of the SARS-CoV-2 virus have emerged. As of March 10, 2023, the World Health Organization has reported approximately 676,609,955 cases and 6,881,955 deaths globally due to SARS-CoV-2 infection^5–7^. Since December 2021, with the emergence of the Omicron variant, the COVID-19 pandemic has entered a new phase. Compared to other Variants of Concern (VOC), Omicron has exhibited a lower mortality rate, leading many countries to adjust their control strategies to this COVID-19 pandemic. China adjusted the public health control measures on December 7, 2022, leading to the beginning of the wide spreading of SARS-CoV-2 infections in the mainland of China.

With the increasing number of the infected individuals, the probability of viral mutations increase. Therefore, to investigate the evolutionary relationship of the SARS-CoV-2 strains is of significance. Moreover, with the diverse range of SARS-CoV-2 strains currently present, it is essential to systematically categorize the available strains, so as to identify the relationship among them and predict the potential strains which may appear in the future. Clustering analysis, particularly the hierarchical clustering analysis, has been extensively applied in phylogenetic studies, including constructing tree, estimating the evolutionary rates, and investigating the gene evolution process^8^. Therefore, this study will employ the hierarchical clustering analysis methods to analyze the relationships among different types of strains of the SARS-CoV-2 virus in China.

We firstly encode the sequences of the SARS-CoV-2 strains using feature encoding algorithm named *k*-mer. Then, four different representative sample selection methods are introduced for selecting a sample from each type of the SARS-CoV-2 strains to comprise the dataset on which to carry out the specific clustering analysis. Subsequently, three hierarchical clustering analysis algorithms named Ward-Euclidean, Ward-Jaccard, and Average-Euclidean are proposed and employed to detect the relationship between the SARS-CoV-2 strains. As a result, we obtain the 12 phylogenetic trees of the SARS-CoV-2 strains.

It was observed that the strains found in China primarily belonged to the Delta and Omicron variants. Results were similar from the data set comprising the representative samples selected using methods 3 and 4. On the contrary, the results are disparate from the dataset of the representative samples selected by methods 1 and 2. Among the employed methods, the most frequently occurring strains are BF.28, BE.1.1.1, BA.5.3, and BA.5.6.4, suggesting that these four strains likely possess distinct characteristics not present in other SARS-CoV-2 strains and may serve as the major sources from which the future strains may evolution. Additionally, attention should be given to strains, such as BA.2.75, CH.1.1, BA.2, BA.5.1.3, BF.7, and B.1.1.214, which exhibit stronger capabilities in terms of immune evasion, transmissibility, and pathogenicity. Their future evolutionary trends should be payed much attention to and monitored closely.

## Data and preprocessing

### Dataset

The dataset utilized in this study was obtained from the global initiative on sharing all influenza data (GISAID) (https://gisaid.org/). This dataset comprises all available genomic sequences of SARS-CoV-2 collected in China till January 7, 2023, including 4551 sequences of 172 types of SARS-CoV-2 viral strains. This dataset comprises two primary files. The first is in TSV (Tab-Separated Values) format, containing information about the viral strains, such as the strain name, collection date and location, and the demographic details of the individuals from whom the samples were collected (such as age and gender). The second file is in FASTA format, containing the actual genetic information of the viral sequences. These sequences are associated with specific strain types. The strain types of the sequences are classified and named using the widely adopted Pangolin lineage assignment method.

This study is to analyze the evolutionary relationships and differences among the existing SARS-CoV-2 strains available in China as of January 7, 2023, utilizing the dataset from the GISAID database. The information provided in the dataset, including strain types and associate metadata, contributed to the comprehensive analysis and understanding of the genetic diversity and characteristics of the SARS-CoV-2 strains circulating within China.

We did not carry out the study on the global dataset of SARS-CoV-2 strains available from the GISAID database because of the very much big volume of it and the limited port speed in our side, which together resulting in its impossible for us to download this global dataset. Moreover, although we narrow our scope to the dataset from China, the types of SARS-CoV-2 strains collected in China till January 7, 2023 cover most of the types of SARS-CoV-2 strains in the global dataset. The relationship between the types of SARS-CoV-2 strains collected in China till January 7, 2023, will not change with the types of SARS-CoV-2 strains in the global dataset. Furthermore, although our dataset may not capture the global diversity of SARS-CoV-2 strains, it provides valuable insights into the relationships of the available SARS-CoV-2 strains and their evolutionary trends.

### Data preprocessing

We performed data preprocessing for two obtained files. For the first file, each sequence sample has two labels, one is the ID in “gisaid_epi_isl” and the other is the type of the strain in “pangolin_lineage”. We classified these data into 172 different strain types based on the labels in “pangolin_lineage”, i.e. with regard to the types of strains.

For the second file, we encode the genetic sequence into numerical values. Since the genetic sequence contains degenerate bases, representing the ambiguity in codons, we replace each degenerate base utilizing one of its corresponding normal bases randomly selected with equal probability. Subsequently, we employ *k*-mer algorithm proposed in^9^, a classic nucleotide sequence feature encoding algorithm, to encode the genetic sequences of the SARS-CoV-2 strains into numerical values. Each sequence will become a numerical vector. Finally, we obtained a feature matrix of 4551 $\times$1364, where each sample consists of 1364 numerical features, and the totally number of samples are 4551. This matrix represents the genetic sequences of SARS-CoV-2 strains in the type of numerical values.

## Methods

This article aims to analyze the relationship between the various types of the SARS-CoV-2 coronavirus strains through conducting hierarchical clustering analysis on all the types of the COVID-19 strains identified in China before January of 2023, so as to speculate on the future mutation and evolution trends of the SARS-CoV-2 coronavirus strains.

We firstly encode the genetic sequences of SARS-CoV-2 coronavirus strains into numerical feature vectors using *k*-mer algorithm proposed in^9^ which is for nucleotide sequence feature encoding. Then, we introduce four methods to select the representative sample of each type of SARS-CoV-2 strain, resulting in four datasets of representative samples of SARS-CoV-2 strains. Finally, the hierarchical clustering analysis is, respectively, performed on these four datasets of representative samples of various types of SARS-CoV-2 strains using the Origin analysis software^10^, while employing both Ward^11^ and Average linkage methods^12^ and the Euclidean and Jaccard distances, respectively. Consequently, three combined methods, named Ward-Euclidean, Ward-Jaccard, and Average-Euclidean, are proposed and employed to perform hierarchical clustering analysis on these four datasets of representative samples of various types of SARS-CoV-2 strains. The clustering results of these three methods are obtained and analyzed.

We do not introduce the Average-Jaccard method to conduct hierarchical clustering analysis on these four datasets of representative samples of various types of SARS-CoV-2 strains, due to the inherent characteristics of the Jaccard distance. If there is not any changes in the Jaccard distance twice in a row, the hierarchical clustering process cannot go on, except for leading to an error reported and the clustering process being terminated. So there are not Average-Jaccard method introduced in this article. The algorithms employed in this study are presented in the following subsections, respectively.

### *k*-mer algorithm

The *k*-mer algorithm proposed by Hersh^9^ is to encode the nucleotide sequences into numerical features. We utilize this classic nucleotide feature encoding algorithm to encode the genetic sequences of the SARS-CoV-2 strains into numerical vectors. Specifically, it breaks down the DNA sequences into overlapping subsequences of length *k* (*k*-mers) and counts the occurrence of each *k*-mer in the sequence. Through representing the frequencies of different *k*-mers, we encode the sequences of the SARS-CoV-2 strains into numerical values for the further clustering analysis and modeling.

The *k*-mer algorithm encodes DNA/RNA sequences by calculating the frequencies of all possible $k\left( 1\le k\le l \right)$ adjacent nucleotides in a sequence in Eq. (1), where *l* is the length of the sequence.

1 $$\begin{aligned} f\left( {{N}_{1}}{{N}_{2}}\ldots {{N}_{k}} \right) =\frac{Count\left( {{N}_{1}}{{N}_{2}}\ldots {{N}_{k}} \right) }{l-k+1} \end{aligned}$$

The $f\left( {{N}_{1}}{{N}_{2}}\ldots {{N}_{k}} \right)$ defined in Eq. (1) represents the frequency of the *k*-mer, i.e., ${{N}_{1}}{{N}_{2}}\ldots {{N}_{k}}$, in the sequence of length *l*, while $Count\left( {{N}_{1}}{{N}_{2}}\ldots {{N}_{k}} \right)$ is the number of occurrences of the *k*-mer in the sequence, and $l-k+1$ is the total count of all *k*-mers in the sequence with length *l*.

There are ${{4}^{k}}$ possible adjacent nucleotides, so a DNA/RNA sequence will be encoded into a numeric feature vector of dimension of ${{4}^{k}}$. The dimensionality of the encoded feature vector becomes exponentially large as the value *k* increases, and the valuable information diminishes for predictive classification tasks. Therefore, the parameter *k* is typically fall in the range from 1 to 5. In this study, the value of *k* ranges from 1 to 5, resulting in the 4, 16, 64, 256, and 1024 features, respectively. These features were combined, resulting in a final feature set of 1364 features.

**Figure 1:**
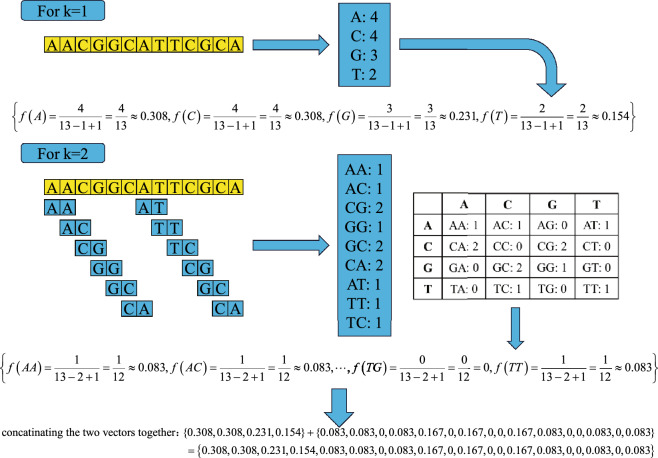
The specific example for encoding a DNA nucleotide sequence into numerical features for $k=1$ and $k=2$.

Here we provide a specific example in Fig. 1 to illustrate the principle of the *k*-mer algorithm. We utilize a DNA nucleotide sequence of length *l* = 13 as an example. That is,  $N_i(i=1,\ldots ,k)\in \left\{ A, C, G, T \right\}$. We only show the encoding process for *k* = 1 and *k* = 2. The process is similar for $k=$ 3, 4, or 5.

Figure 1 demonstrates how the *k*-mer algorithm works. For $k=1$, we count the occurrence number of *A*,  *C*,  *G* *and* *T* in this example sequence of length 13, respectively; then the frequency of each single nucleotide is calculated in Eq. (1), and we obtain the values of *f*(*A*),  *f*(*C*),  *f*(*G*), *and* *f*(*T*). Similarly, for $k=2$, we first count the occurrence numbers of 16 types of dinucleotides of  $AA,~AC, ~\ldots , ~TA, ~\ldots , ~TT$ in this sequence, then we calculated the frequencies of these 16 types of dinucleotides in this sequence in Eq. (1), and obtained the value of each of $f(AA),~f(AC),~f(AG), ~f(AT),~f(CA),~\ldots ,~f(TT)$.

It should be noted that there are only 9 dinucleotides, including *AA*,  *AC*,  *CG*,  *GG*,  *GC*,  *CA*, *AT*, *TT* and *TC*, appearing in the sequence of this example. The rest 7 dinucleotides do not appear in this sequence, so the frequencies of them are 0. Figure 1 also show how the numerical vectors for *k* = 1 and *k* = 2 are concatenated together as the one numerical vector.

### DPC algorithm

The density peak clustering (DPC) algorithm is a novel density based clustering algorithm, proposed by Rodriguez and Laio in 2014^13^. It can identify clusters in a dataset based on the densities of data points. It offers a simple yet effective way to discover clusters without requiring the number of clusters as input parameter. It assumed that the cluster centers usually have higher density than their neighbors, and the data point with higher density usually far away from each other. The most significant definitions in DPC algorithm is the local density and distance of a point. The local density $\rho _{i }$ of point i is calculated in Eq. (2):

2 $$\begin{aligned} {{\rho }_{i}}=\sum \nolimits _{j\ne i}{\chi \left( {{d}_{ij}}-{{d}_{c}} \right) } \end{aligned}$$

where the ${{d}_{ij}}$ is the Euclidean distance between points *i* and $j\left( i,j=1,\ldots ,N \right)$ , and *N* is the number of points in the dataset, and ${{d}_{c}}$ is the *cutoff* distance that is the threshold to calculate the local density of point *i*, and $$\chi \left( x \right) =\left\{ \begin{aligned}&1,x<0 \\&0,x\ge 0 \\ \end{aligned} \right.$$. This definition indicate the local density ${{\rho }_{i}}$ of point *i* counts the number of points around it within the neighborhood with the radius of ${{d}_{c}}$. The local distance ${{\delta }_{i}}$ of point *i* is calculated in Eq. (3):

3 $$\begin{aligned} {{\delta }_{i}}=\left\{ \begin{aligned}&\underset{{{\rho _i} ={\mathop {\max }}\{\rho _j\}}}{\mathop {\max }}\{d_{ij}\}\\&\underset{j:{{\rho }_{j}}>{{\rho }_{i}}}{\mathop {\min }}\, \{{d}_{ij}\}\\ \end{aligned} \right. \end{aligned}$$

The novel contribution of DPC is the decision graph which is constructed using local density $\rho$ as *x*-axis and local distance $\delta$ as *y*-axis, so that each data point is represented as a node in the two-dimensional space referred to as decision graph. The points in the upper right corner with both higher local density and distance are density peaks, comprising Cluster Centers. They are far away from those in the lower left corner.

After the cluster centers have been detected out, the non-density peaks are to be assigned to the same cluster as the nearest point with higher local density. By utilizing the local density and distance information, the DPC algorithm is capable of identifying clusters with any arbitrary shapes and sizes without requiring the number of clusters as an input parameter. It has been shown to be effective in various applications, including image segmentation^14^, pattern recognition, and data mining^15^.

### Representative sample selection methods

We perform hierarchical clustering analysis to the genomic sequences of the SARS-CoV-2 strains, so that we can detect out the evolutionary relationships between the various types of the SARS-CoV-2 strains. However, too large number of samples in some categories of the SARS-CoV-2 strains will significantly affect the clustering results, making it difficult to obtain the accurate hierarchical clustering result to represent the evolutionary relationships between the types of the SARS-CoV-2 strains. Hence, we propose methods to select a representative sample (centroid) from each category of the SARS-CoV-2 strains to comprise the dataset on which to apply the hierarchical clustering analysis to detect the evolutionary relationships among the various types of the SARS-CoV-2 strains. Four methods are proposed in this study to find the representative samples. They are described here .

1. The mean of all samples within each category is calculated, resulting in a new sample (centroid) of each category, serving as the representative sample for that category. This process can be described as followings. Assume that there is *K* categories within a data set, and category $i(i=1,\ldots ,K)$ containing $n_i$ samples. The samples in category *i* comprise a subset $\textbf{X}^{i}=\left\{ \textbf{x}^i_{k}|k=1,\ldots ,n_i \right\}$, and $\textbf{x}^i_k=(x^i_{1k},\ldots ,x^i_{jk},\ldots , x^i_{mk})$, where *m* represents the number of features of a sample. Then we compute the average of all samples in each category. Such as the average of feature *j* of all samples in category *i* is:
  4 $$\begin{aligned} {{\mu }^{i}_{j}}=\frac{1}{{{n}_{i}}}\sum \limits _{k=1}^{{{n}_{i}}}{{{x}^i_{jk}}} \end{aligned}$$
  After that, we obtain the centroid $C_i$ of category *i*, which is the following vector containing the average value of each feature of all samples within category *i*.
  5 $$\begin{aligned} {{\textbf{C}}_{i}}=\left( {{\mu }^{i}_{1}}, \ldots , \mu ^i_j,\ldots ,{{\mu }^{i}_{m}} \right) \end{aligned}$$
  In this way, for each category, the average values of features are calculated, resulting in the centroid for that category, which serves as the representative sample of that category.
2. The DPC algorithm is utilized to detect the most representative sample from each category of the SARS-CoV-2 strains. We compute the local density and distance of each sample, as well as the product of the local density and distance called gamma of a sample. For each category, the sample with the highest gamma is chosen as the representative sample for that category.
3. The sample closest to the centroid in terms of Euclidean distance is selected as the representative sample of the category covering this centroid. This process can be expressed in detail through the following mathematical description: first, compute the Euclidean distance between a sample and the centroid of that category containing this sample. The Euclidean distance from sample *k* in category *i* to its centroid is calculated by:
  6 $$\begin{aligned} {{d}_{C_ik}}=\sqrt{\sum \limits _{j=1}^{m}{{{\left( {{x}^{i}_{jk}}-{{\mu }^{i}_{j}} \right) }^{2}}}} \end{aligned}$$
  Here, $\mu ^{i}_{j}$ is the average value of feature *j* of all samples within category *i*, that is, the element *j* of centroid of category *i*. Then, select the sample closest to the centroid as the representative sample. For example, for category *i*, sample *p* will be selected as the representative sample if it satisfies the following condition:
  7 $$\begin{aligned} p=\arg {{\min }^{n_i}_{k=1}}{{d}_{C_ik}} \end{aligned}$$
  Thus, each category will have one sample chosen as the representative sample, which is the sample closest to its centroid.
4. The sample minimizing the total Euclidean distance to other samples within same category is chosen as the representative sample of that SARS-CoV-2 strain category. This involves calculating the sum of Euclidean distances from a sample to the other samples within same category and selecting the sample with the smallest sum of the Euclidean distances. This process can be described in detail through the following mathematical description. Assume that there are $n_i$ samples in category *i*. The subset of samples in category *i* is denoted as $\textbf{X}^{i}=\left\{ \textbf{x}^i_{k}|k=1,\ldots ,n_i \right\}$, and $\textbf{x}^i_k=(x^i_{1k},\ldots ,x^i_{jk},\ldots , x^i_{mk})$, where *m* represents the number of features of a sample. Then, the Euclidean distance between each sample and other samples in same category is computed. For example, let the Euclidean distance between samples *p* and *q* be denoted as $d_{pq}$, then for sample *p* in category *i*, the total Euclidean distances from *p* and to all other samples *q* within same category is calculated as:
  8 $$\begin{aligned} {{D}^i_{p}}=\sum \limits _{q=1,q\ne p}^{{{n}_{i}}}{{{d}_{pq}}} \end{aligned}$$
  After that, the sample minimizing the total distance is selected as the representative sample of category $i(i=1,\ldots ,K)$, that is, we select sample $p^*$ which satisfies the following condition as the representative sample for category *i*.
  9 $$\begin{aligned} {{p}^{*}}=\arg {{\min }^{n_i}_{p=1}}{{D}^i_{p}} \end{aligned}$$

### Proposed hierarchical clustering algorithms

The hierarchical clustering algorithm constructs a hierarchical nested clustering tree by computing the similarities between data points^16^. In the clustering tree, the original data points locate at the bottom layer, while the top layer represents the root node of the clustering tree. In this study, we employed two hierarchical clustering algorithms named Ward^11^ and Average^12^.

The Ward clustering algorithm, also known as Ward’s minimum variance algorithm, is a hierarchical clustering algorithm that aims to minimize the within-cluster variance when merging clusters. It was proposed by Joe Ward in 1963^11^. The Ward algorithm follows a rule of merging the pair of clusters with the smallest distance between points from them. On the other hand, the Average algorithm defines the distance between two clusters as the average distance between each point in one cluster and every points in the other cluster. It then merges the pair of clusters with the smallest average distance.

The Ward algorithm starts with each point as an individual cluster. It iteratively merges the most appropriate pair of clusters to form the hierarchical structure of points within a dataset. At each merging step, it determines the pair of clusters whose merging will result in the smallest increase in the total within-cluster variance. This increase in variance is measured by the difference of the sum of squared Euclidean distances between data points and their cluster centroids of the clustering before and after merging.

The Ward algorithm utilizes a bottom-up approach to detect the hierarchical structure hidden in a datasets. This type of hierarchical clustering algorithm is also known as agglomerative clustering algorithm. It repeatedly combines the closest pair of clusters until all data points belonging to a single cluster. The distance between clusters can be calculated using various methods, such as Euclidean distance or squared Euclidean distance, etc.. In this study, the Euclidean and Jaccard distances are utilized in Ward algorithm.

The idea of Ward algorithm is as follows. Given data set $\textbf{X} = \{\textbf{x}_1, \textbf{x}_2,\ldots , \textbf{x}_n\}$, and let *AB* denote the cluster obtained by merging clusters *A* and *B*. We compute the difference between the sum of squared variances before and after merging clusters *A* and *B*. The sum of squared variances before and after merging clusters *A*, *B* are, respectively, as follows:

10 $$\begin{aligned} SS{{E}_{A}}= & {} \sum \limits _{i=1}^{{n}_{A}}{{\left( {\textbf{x}_{i}}-\bar{\textbf{x}}_{A} \right) }^{\prime }}\left( {\textbf{x}_{i}}-\bar{\textbf{x}}_{A} \right) \end{aligned}$$

11 $$\begin{aligned} SS{{E}_{B}}= & {} \sum \limits Wa_{i=1}^{{{n}_{B}}}{{\left( {\textbf{x}_{i}}-\bar{\textbf{x}}_{B} \right) }^{\prime }}\left( {\textbf{x}_{i}}-\bar{\textbf{x}}_{B} \right) \end{aligned}$$

12 $$\begin{aligned} SS{{E}_{AB}}= & {} \sum \limits Wa_{i=1}^{{{n}_{AB}}}{{\left( {\textbf{x}_{i}}-\bar{\textbf{x}}_{AB} \right) }^{\prime }}\left( {\textbf{x}_{i}}-\bar{\textbf{x}}_{AB} \right) \end{aligned}$$

where $n_A$ and $n_B$ represent the number of elements in clusters *A* and *B*, respectively. The ${\bar{\textbf{x}}_A}=\frac{\sum \nolimits _{i=1}^{{{n}_{A}}}{{\textbf{x}_{i}}}}{{{n}_{A}}}$, ${\bar{\textbf{x}}_B}=\frac{\sum \nolimits _{i=1}^{{{n}_{B}}}{{\textbf{x}_{i}}}}{{{n}_{B}}}$, and $\bar{\textbf{x}}_{AB} = \frac{\sum _{i=1}^{n_A} \textbf{x}_i + \sum _{j=1}^{n_B} \textbf{x}_j}{n_A + n_B} = \frac{n_A \bar{\textbf{x}}_A + n_B \bar{\textbf{x}}_B}{n_A + n_B}$ are,respectively, the mean of clusters *A*, *B* and *AB*.

This algorithm starts from each point in $\textbf{X}$ is as a single cluster. The following process is repeated till all points in a same cluster. For all pairs of clusters, clusters *A* and *B* are merged if $SSE_{AB} - (SSE_A + SSE_B)$ is minimal, indicating merging cluster *A* and *B* resulting in the smallest increment in the sum of squared errors of the clustering. The Ward algorithm has several advantages. It tends to produce compact and balanced clusters, making it suitable for datasets with varying sizes and shapes of clusters. Additionally, it is less sensitive to initial conditions compared to other clustering algorithms.

However, the Ward algorithm can be computationally expensive for large datasets due to its comparative high time complexity. It is commonly used in exploratory data analysis, pattern recognition, and clustering applications where minimizing within-cluster variance is a desired objective. We utilized it because we did clustering analysis to the genomic sequences of the SARS-CoV-2 strains through the representative samples of the various types of them.

The Average algorithm defines the distance between two clusters as the average distance between each point in one cluster and every points in the other cluster. It then proceeds to merge the pair of clusters with the smallest average distance. The average hierarchical clustering method is a technique used in hierarchical clustering to determine the distance between clusters. It is also known as average linkage clustering or UPGMA (Unweighted Pair Group Method with Arithmetic Mean). It was introduced by Sokal and Michener in 1958^12^.

In the Average linkage method, the distance between two clusters is defined as the average of the pairwise distances between all pairs of data points from two clusters. This average distance is calculated using a suitable distance metric, such as Euclidean distance or Manhattan distance, etc. In this study, the Euclidean distance is utilized. The algorithm starts with each data point as an individual cluster and then progressively merges the closest pair of clusters based on their average distance until all data points are grouped into a single cluster.

The idea of Average hierarchical clustering algorithm is as follows. Given data set $\textbf{X} = \{\textbf{x}_1, \textbf{x}_2, \ldots , \textbf{x}_n\}$, and a distance matrix $\textbf{D}$ measuring the Euclidean distance between data points in $\textbf{X}$. Average hierarchical clustering process starts from each data point is as a cluster $\textbf{C}_i={\textbf{x}_i}$. Then, the average distance between any two clusters, such as clusters *A* and *B*, is calculated as follows:

13 $$\begin{aligned} {{{d}_{avg}}\left( {A},{B} \right) =\frac{1}{{{n}_{A}}\times {{n}_{B}}}\sum \nolimits _{i=1}^{{{n}_{A}}}{\sum \nolimits _{j=1}^{{{n}_{B}}}{d_{ij}}}} \end{aligned}$$

Here, $n_A$ and $n_B$ represent the number of points in clusters *A* and *B*, respectively, and $d_{ij}$ denotes the distance between the point *i* of cluster *A* and the point *j* of cluster *B*. The two clusters *A* and *B* will be merged together as one cluster if and only if the $d_{avg} (A,B)$ is the smallest among all the average distances between current pairs of clusters. Unlike other linkage methods, such as complete linkage or single linkage, Average linkage method considers the average distance between clusters rather than the minimum or maximum distance. This leads to more balanced and less biased clustering results, particularly in cases where complete linkage or single linkage will produce elongated or uneven size of clusters.

Average linkage method is commonly used in various applications, including biology, genetics, and social sciences. It is particularly useful when the dataset contains noises or outliers that could affect the clustering results, because the average distance calculation is less sensitive to extreme values. However, it should be noted that Average linkage method is sensitive to the presence of outliers with large distances, which can impact the overall clustering structure.

In addition, the Ward and Average linkage methods adopted in this study exhibit strong interpretability to clustering results. The Ward method constructs the hierarchical clusters by minimizing the within-cluster variance, resulting in the more compact and consistent clusters that align well with the human’s understanding of clustering outcomes. On the other hand, the Average linkage method determines clusters by computing the average distance between samples from two clusters, respectively, providing intuitive explanations. The hierarchical clustering tree generated by Ward or Average linkage method can be visualized using appropriate tools, facilitating a clear representation of the similarity between samples and the clustering structure hidden within samples. This can aid researchers to understand and interpret the clustering results better, as well as discovering the underlying patterns and trends hidden within a dataset.

### The idea of the overall algorithm

**Figure 2:**
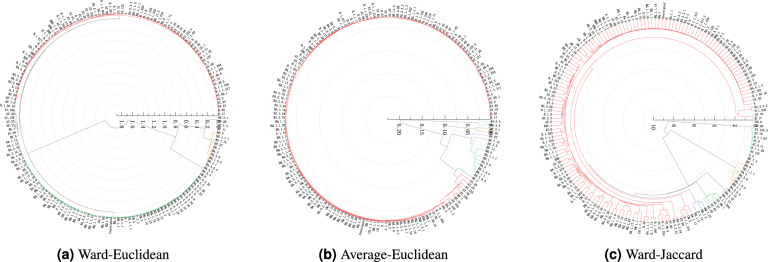
The pedigree diagrams of the existing types of the SARS-CoV-2 virus strains obtained through three hierarchical clustering methods of Ward-Euclidean, Average-Euclidean and Ward-Jaccard, respectively. The representative samples are selected utilizing method (1) proposed in “Representative sample selection methods”.

**Figure 3:**
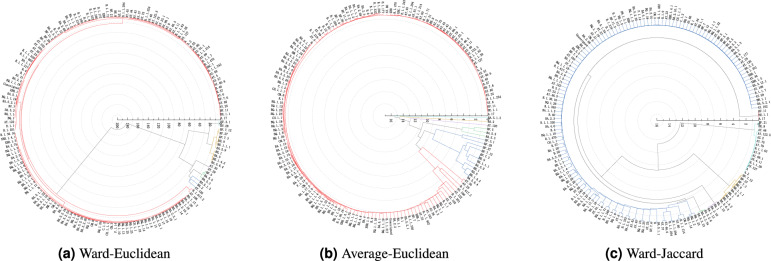
The pedigree diagrams of the existing types of the SARS-CoV-2 virus strains obtained through three hierarchical clustering methods of Ward-Euclidean, Average-Euclidean and Ward-Jaccard, respectively. The representative samples are selected utilizing method (2) proposed in “Representative sample selection methods”.

**Figure 4:**
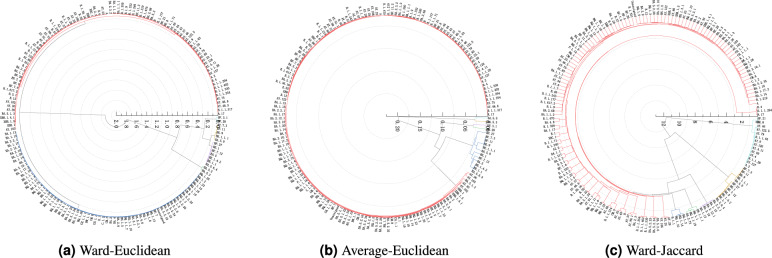
The pedigree diagrams of the existing types of the SARS-CoV-2 virus strains obtained through three hierarchical clustering methods of Ward-Euclidean, Average-Euclidean and Ward-Jaccard, respectively. The representative samples are selected utilizing method (3) proposed in “Representative sample selection methods”.

**Figure 5:**
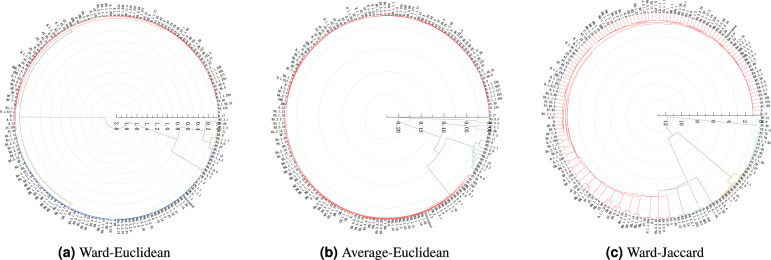
The pedigree diagrams of the existing types of the SARS-CoV-2 virus strains obtained through three hierarchical clustering methods of Ward-Euclidean, Average-Euclidean and Ward-Jaccard, respectively. The representative samples are selected utilizing method (4) proposed in “Representative sample selection methods”.

This section will summarize the overall method introduced in this paper for conducting hierarchical clustering analyses to the available SARS-CoV-2 virus strains while investigating the evolutionary relationships between the various types of the SARS-CoV-2 strains utilizing the genomic sequences. The idea of the overall algorithm designed in this article is described as follows:

Step 1): The *k*-mer algorithm described in section *k*-*mer algorithm* is utilized to encode the genetic sequences of the SARS-CoV-2 coronavirus strains into numerical feature vectors.
Step 2): The four representative sample selection methods proposed in section *Representative sample selection methods* are employed to select the representative sample from each type of SARS-CoV-2 virus strains’ numerical feature vectors encoded by Step 1) to comprise the four representative sample datasets of SARS-CoV-2 virus strains for subsequently conducting hierarchical clustering analysis.
Step 3): The Ward-Euclidean, Ward-Jaccard, and Average-Euclidean hierarchical clustering algorithms proposed in section *Proposed hierarchical clustering algorithms* are employed to perform hierarchical clustering analysis through OriginPro software on four representative sample datasets obtained using Step 2), so as to obtain the corresponding clustering results shown in Figs. 2, 3, 4 and 5. The representative type of SARS-CoV-2 virus strains of the clustering results are displayed in Table 1.

## Experiments and analyses

We first encode the genomic sequences of SARS-CoV-2 strains into numerical data, then select the representative sample of each type of SARS-CoV-2 strains to comprise the datasets on which to perform the hierarchical clustering analysis for detecting the evolutionary relationship between the available types of the SARS-CoV-2 strains by constructing the corresponding dendrograms of the SARS-CoV-2 strains. There are four methods employed to select representative samples, and three hierarchical clustering algorithms such as Ward-Euclidean, Ward-Jaccard, and Average-Euclidean are employed to conduct clustering analysis. The OriginPro software is adopted in our experiments. Figures 2, 3, 4 and 5 demonstrate the hierarchical clustering results corresponding to the four representative sample selection methods proposed in “Representative sample selection methods”. Table 1 displays the specific SARS-CoV-2 virus strain of each cluster depicted in Figs. 2, 3, 4 and 5. It should be noted that the specific SARS-CoV-2 virus strain of a cluster is the one that exhibits the minimum sum distance to all other strains within the same cluster.

We display the clustering results in circular dendrograms to represent the relationships and similarities among different types of the SARS-CoV-2 virus strains. Each type of virus strain is depicted as a node, and the connections between the nodes indicate the distances and similarities between them. The positioning of nodes is based on the genetic relatedness within the dendrograms. By examining the distributions and connectivity patterns of the nodes in a dendrogram displayed in Figs. 2, 3, 4 and 5, we can infer the genetic relationships and evolutionary paths among the existing types of the SARS-CoV-2 virus strains.

The clustering results in Fig. 2a,b are both based on the Euclidean distance, so they are quite similar to each other, such as the strains of BA.5.5 and BA.5.3 are clustered together in both of the clustering results depicted in Fig. 2a,b, so do the strains of BF.3.1 and BF.28. Moreover, the results shown in Fig. 2b demonstrate that BF.27 comprises a separate cluster firstly, then it is grouped with BA.5.6.4, BE.1.1, BE.1.4.2, BA.5, BA.5.9, BE.1, BE.1.1.1, BE.9, BF.11, BF.7.3, and BF.7.12 together to comprise a larger cluster. The results shown in Fig. 2b also demonstrate that BF.5 constructs a separate cluster firstly, then it is grouped with all of the remaining strains except for aforementioned ones, followed by merging with the aforementioned clustering branches comprising BF.27, BF.28 and BA.5.3, respectively, one by one until the final hierarchical clustering result is obtained comprising all of the SARS-CoV-2 virus strains.

The results displayed in Fig. 2c show that BN.1.5, P.1.14, BQ.1.2, and BQ.1.23 are clustered together, then they are grouped with the majority of the existing strains marked in red in Fig. 2c, such as A.17 et cetera. After that they are grouped with AY.43, AY.44, AY.57, AY.99.1, and B.1.1.294. Next, they are clustered together with BA.1.13, BA.2.10, and BA.1.15. Following, they are grouped with BA.5.6.4, BE.1.1, BE.1.4.2, BE.1, BA.5.9, BA.5, BE.1.1.1, BE.9, BF.11, and BF.7.3. Finally, they are merged with the cluster comprising A.2, AY.127, B.1.1.28, AY.4.11, AY.9, B.1.1.63, AY.79, AY.122.5, AY.46, and BE.8 strains into final one cluster.

The results displayed in Fig. 3a demonstrate that BF.3.1 and BF.28 are first clustered together, then they are grouped with BA.5.6.4, BE.1.4.2, BA.5, BE.1, BA.5.9, BE.1.1.1, BE.9, BF.7.3, BF.27, and BF.7.12 strains. The strains of AY.126, BA.2, and BA.5.1.3 are clustered together first, then they are grouped with the cluster comprising BA.5.3, BA.5.5, and BF.5 strains. After that they are cluster together with the cluster comprising AY.29 and AY.30 strains. Finally they are clustered with the cluster comprising the aforementioned strains, such as BA.5.6.4 and BF.3.1, et cetera, and subsequently with all of the remaining strains together as the final one cluster.

The results presented in Fig. 3b demonstrate that BA.5.1.3, BA.2, and AY.126 constitute separate clusters by each of them. The strains of BF.5 and BA.5.5 are grouped together first, then they are grouped with BA.5.3 into one cluster. The strains of BF.28 and BF.3.1 are grouped as one cluster. The strains of BE.1.1.1, BE.9 and BF.7.3 are first grouped together, then they are grouped with BA.5.9, BF.7.12 and BF.27 in turn. Next, they are grouped with the cluster, where the strains BA.5.6.4 and BE.1.4.2 are first grouped together, then are grouped with BA.5 and BE.1 in turn. After that, this new cluster is grouped together with the cluster comprising BF.28 and BF.3.1. Finally, these aforementioned clusters are grouped together with the majority of strains marked in red in Fig. 3b, including A.17, AY. 30, etcetera.

The results presented in Fig. 3c demonstrate that BA.5.2.6, BA.5.2.34, and BA.5.2.23 are first clustered together as one cluster, then they are grouped with the majority of strains for SARS-CoV-2 virus marked in blue within Fig. 3c, including AY.102, BQ.1.23, etcetera. After that, they are grouped with A.17, BE.1.1, and BF.11. Next, they are together clustered with BA.1.13, BA.2.10, BA.1, and BA.1.15 together. They are then grouped with BA.5.6.4, BE.1.4.2, BA.5, BE.1, BA.5.9, BE.1.1.1, BE.9, BF.7.3, BF.27, and BF.7.12 strains. Finally, they are merged with AY.127, B.1.1.28, AZ.2, BE.8, BF.21, A.2 and the total of 15 strains into final one cluster.

The results shown in Fig. 4a demonstrate that BE.1.1.1, BE.9, BF.11, and BF.7.3 are first clustered together with BA.5.9, then they are grouped together with BA.5.6.4, BE.1.1, BE.1.4.2, BA.5, BF.7.12, BE.1, and BF.27 strains. Then they are grouped with the cluster comprising BF.3.1 and BF.28 strains. Next, they are clustered with the cluster comprising BA.5.5 and BA.5.3 strains. Finally, they are merged with all remaining SARS-CoV-2 virus strains into one cluster, though these remaining trains of SARS-CoV-2 virus are grouped as two clusters colored in blue and red, respectively, in Fig. 4a.

The results presented in Fig. 4b demonstrate that BF.28, BF.3.1, BA.5.3, and BA.5.5 constitute clusters separately. The strains of BA.5.6.4, BA.5, BE.1.1, BE.1.4.2, BF.7.12, BE.1.1.1, and 12 other strains of SARS-CoV-2 virus are first clustered together, then they are grouped with all of the remaining strains except for BF.28, BF.3.1, BA.5.3, and BA.5.5. Then they are grouped together with the cluster constituted by BF.3.1 and BF.28. Finally, they are clustered into same one cluster with the cluster constituted by BA.5.5 and BA.5.3 strains of SARS-CoV-2 virus.

The results shown in Fig. 4c demonstrate that the majority of strains of SARS-CoV-2 virus marked in red, including A.17, AY.29, and et cetera are first clustered together, then they are grouped together with the cluster comprising of B.1.1, BQ.1.10, BQ.1.14, and BQ.1.2 strains. Next they are grouped with the cluster comprising of BA.2.75, BQ.1.1.19, BQ.1.1, and BN.1.1.1 strains together. Followed, they are clustered with the cluster comprising of BA.1.13, BA.2.10, BA.2.12.1, BA.1.15, and BA.2.2.1 together as same one cluster. After that they are grouped together with the cluster comprising of BA.5.6.4, BE.1.1, BE.1.4.2, BA.5, BF.7.12, BE.1, BF.27, BA.5.9, BE.1.1.1, BE.9, BF.11 and BF.7.3 strains into one cluster. Finally, this newly constructed cluster is merged with the cluster comprising 14 strains of SARS-CoV-2 virus including A.2, BF.21, and et cetera to constitute the final one cluster of SARS-CoV-2 virus strains.

The results depicted in Fig. 5a demonstrate that BA.5.6.4, BA.5, BE.1.1, BE.1.4.2, BF.7.12, BE.1, and BF.27 are first clustered together, then they are grouped with the cluster comprising strains of BA.5.9, BE.1.1.1, BE.9, BF.11, and BF.7.3 strains. Then they are grouped with the cluster comprising strains of BF.3.1 and BF.28. Next, they are grouped together with the cluster comprising BA.5.5 and BA.5.3 strains. Finally, they are merged with the cluster comprising two clusters constituted by all remaining unmentioned strains into the final one cluster.

The results depicted in Fig. 5b demonstrated that this clustering results are consistent with the clustering results depicted in Fig. 4b, with only a slight difference in the order of clustering.

The results depicted in Fig. 5c demonstrate that BA.1.13, BA.2.10, BA.1.15, and BA.2.2.1 strains are first clustered together, then they are grouped with the cluster comprising of the majority of strains marked in red, including A.17, AY.44, and et cetera. Then they are grouped together with the cluster comprising of four strains starting with the B.1.1 (including B.1.1.254, B.1.1.317, B.1.1.402, and B.1.1.330). Next, they are clustered together with the cluster comprising strains of BA.2, BA.5.1.24, BA.5.1.3, and BA.5.1.22 strains. After that, they are grouped together with the cluster comprising of the 12 strains of BA.5.6.4, BA.5, BE.1.1, BE.1.4.2, BF.7.12, BE.1, BF.27, BA.5.9, BE.1.1.1, BE.9, BF.11 and BF.7.3 strains. Finally, they are merged with 14 strains including AY.127, BF.21, and et cetera strains into final one cluster.

From the aforementioned hierarchical clustering results depicted in Figs. 2, 3, 4 and 5 with regard to the genomic sequences of the SARS-CoV-2 strains collected in China till January 7, 2023, we can conclude that these strains can be classified into 6 clusters. Table 1 displays the representative strain of each cluster detected by the OriginPro software for the SARS-CoV-2 coronavirus, based on the hierarchical clustering results depicted in Figs. 2, 3, 4 and 5. The detail of the available strains can be found in Table S1 in Supplementary materials.

**Table 1:** The specific SARS-CoV-2 strain of each cluster returned by OriginPro software from the clustering depicted in Figs. 2, 3, 4 and 5 of the available SARS-CoV-2 strains.

Method for selecting representative sample (related results)	Clustering algorithm	Cluster 1	Cluster 2	Cluster 3	Cluster 4	Cluster 5	Cluster 6
Method (1)	Ward-Euclidean	AY.57	BA.5	BF.28	BA.5.3	BF.7	BA.2.75
(results in Fig. 2)	Average-Euclidean	BF.27	BA.5	BF.28	BA.5.3	BF.5	BA.5.2
	Ward-Jaccard	AY.57	BE.1.1.1	AY.127	BA.1.13	BN.1.5	B.1.1.214
Method (2)	Ward-Euclidean	A.17	BE.1.1.1	AY.126	BA.5.5	AY.29	BF.28
(results in Fig. 3)	Average-Euclidean	A.17	BE.1.1.1	AY.126	BA.5.5	BA.2	BA.5.1.3
	Ward-Jaccard	A.17	BE.1.1.1	A	BA.5.2.34	BA.2.10	BQ.1.14
Method (3)	Ward-Euclidean	BA.5.6.4	BA.5.3	BF.28	A.17	BE.1.1.1	CH.1.1
(results in Fig. 4)	Average-Euclidean	BA.5.6.4	BA.5.3	BF.28	BA.5.5	BF.3.1	BQ.1.1.18
	Ward-Jaccard	BA.5.6.4	BA.2.2.1	AY.127	AY.29	BQ.1.14	BQ.1.1.19
Method (4)	Ward-Euclidean	BA.5.6.4	BA.5.3	BF.28	AY.30	BE.1.1.1	CH.1.1
(results in Fig. 5)	Average-Euclidean	BA.5.6.4	BA.5.3	BF.28	BA.5.5	BF.3.1	BQ.1.1.18
	Ward-Jaccard	BA.5.6.4	BA.2.2.1	AY.127	AY.4	BA.2	B.1.1.317

The results depicted in Table 1 demonstrate that there are three common strains in the clustering results depicted in Fig. 2a,b, which is obtained through Ward-Euclidean and Average-Euclidean clustering algorithms, respectively. They are BA.5, BF.28, and BA.5.3 strains from cluster 2, cluster 3, and cluster 4, respectively. Conversely, the differing three strains in other three clusters are AY.57, BF.7, and BA.2.75 in Fig. 2a compared to BF.27, BF.5, and BA.5.2 in Fig. 2b. However, the hierarchical clustering depicted in Fig. 2c, utilizing Ward-Jaccard algorithm, shows minimal similarity to that utilizing the aforementioned two clustering algorithms of the Ward-Euclidean and Average-Euclidean depicted in Fig. 2a,b, respectively. The only common strain between the clustering results of Fig. 2a,c is the AY.57 strain presenting the cluster 1 of them, respectively. They employed the same hierarchical clustering method (Ward) but with Euclidean and Jaccard distance, respectively. The five strains of the remaining five clusters of the clustering results depicted in Fig. 2c are BE.1.1.1, AY.127, BA.1.13, BN.1.5, and B.1.1.214 strains.

Considering the SARS-CoV-2 strains obtained through utilizing the Ward-Euclidean algorithm combining Method (1) among four representative sample selection methods, that is, the clustering results depicted in Fig. 2a, the AY.57 strain corresponds to the Delta variant, while the other five strains represent the Omicron variants. The AY.57 strain exhibits substantial dissimilarities in sequence features compared to other five strains, displaying unique mutations absent in the other variants, and vice versa. Among the other five strains, BA.2.75 stands out with significant differences, such as additional mutations in the N-terminal domain but lacking two crucial mutations (L452R and F486V) in the receptor-binding domain (RBD). Despite accumulating the highest number of mutations in the spike protein, BA.2.75 still demonstrates weaker serum escape ability compared to the BA.5 strain^17^.

Moreover, the BA.5, BF.28, BA.5.3, and BF.7 strains possess L452R and F486V mutations that are absent in BA.2.75, contributing to the increased transmissibility and evasion of the immune system^18^. The L452R mutation at the spike protein site enhances infectivity, while the F486V mutation assists the virus in evading the immune system. Furthermore, BA.2.75 and BA.5 strains share an additional mutation, R493Q, in the spike protein^19^. Additionally, BA.2.75 has G446S and N460K mutations which are not present in the other variants. The G446S and R493Q mutations enable the variant to evade antibodies, including those induced by vaccination and previous infection, while R493Q enhances BA.2.75’s ability to bind to human ACE2 receptors^20^. In addition, G446S has been predicted as a potential escape site for antibodies induced by current vaccines against Omicron^21^. The N460K mutation on the spike protein enhances resistance to neutralizing antibodies, aids immune evasion, and facilitates faster antigen receptor binding^22^. The higher binding affinity of BA.2.75 confers an advantage over BA.5^23^. Besides these mutations, BA.2.75 exhibits P1640S, S1221L, and N4060S mutations in ORF1a, a G662S mutation in ORF1b, and a T11A mutation in the E protein^20^. These amino acid substitutions are speculated to enhance the efficiency of the viral RNA replication and contribute to the widespread dissemination of SARS-CoV-2 coronavirus.

Now, let’s shift our focus to the BF.7 variant. BF.7 can be traced back to the Omicron variant known as BA.5. It is a highly pathogenic strain with a shorter incubation period. Moreover, it has a higher potential for reinfection, even among vaccinated individuals^24^. Compared to other Omicron subvariants, Omicron BF.7 possesses a genetic change (R346T) in the receptor-binding site, absent in the other five strains^25^. The symptoms caused by BF.7 are similar to those caused by other Omicron subvariants. Symptoms of infections may include fever, cough, sore throat, runny nose, fatigue, vomiting, and diarrhea. Additionally, other symptoms may comprise nasal congestion, hoarseness, exhaustion, and phlegm. Individuals with compromised immune systems are at a higher risk of developing severe illness when exposed to this subvariant^24^.

In terms of timeline, the BA.5.3, BF.7, and BA.2.75 strains all started to emerge in the early of 2022 and continue to be observed till current time. On the other hand, AY.57 first appeared in the early of 2021 but was last detected in June of 2022. The BF.28 and BA.5 strains emerged around the same time in July of 2020 and are still being observed. The specific appearing time of each strain can refer to Table S1 in the Supplementary materials.

The results shown in Table 1 demonstrate that all variants obtained by Average-Euclidean algorithm with representative sample selection Method (1), that is, the hierarchical clustering results of Fig. 2b, belong to the Omicron lineage. Among them, BF.27 has additional mutations A2784V in ORF1a and A65D in ORF8, and BF.28 has an additional Q44L mutation in ORF1a, while BA.5.3 has an additional Q556K mutation in ORF1a. The strain of BF.5 exhibits an extra A1020S mutation in the Spike protein and an additional H47Y mutation in ORF7a compared to the other five variants. Additionally, the BA.5.2 strain, unique to the variants circulating in China, has an additional T1050N mutation in ORF1b^26^ compared to the other five variants.

From a chronological perspective, BF.27 and BA.5.3 both started to appear in the early of 2022. On the other hand, BF.28, BF.5, BA.5, and BA.5.2 all emerged in the July of 2020 and continue to be observed till recently. For the specific information, please refer to Table S1 in the Supplementary materials.

Among the strains presented in Table 1 obtaining through the Ward-Jaccard algorithm combining the Method (1) for selecting representative samples, that is, of the clustering results depicted in Fig. 2c, the AY.57 and AY.127 strains belong to the Delta lineage, while BE.1.1.1, BA.1.13, and BN.1.5 are classified as Omicron variants. As for B.1.1.214, the major distinction from the other five variants lies in a single mutation, D614G, in the Spike protein. Outside the Spike protein, the B.1.1.214 strain exhibits specific mutations: N: M234I, NSP14:P43L, and NSP16: R287I. It is speculated that these amino acid substitutions enhance the efficiency of the viral RNA replication, contributing to the widespread transmission of SARS-CoV-2 coronavirus^27^.

The results of Method (2) for selecting the representative samples presented in Table 1 show us that when using the Euclidean distance, the hierarchical clustering algorithms of Ward-Euclidean and Average-Euclidean produced similar results in terms of the six clusters they obtained from the existing types of SARS-CoV-2 strains. Among the results of Ward-Euclidean and Average-Euclidean algorithms, there are four clusters consistent, specifically, the A.17, BE.1.1.1, AY.126, and BA.5.5 are the common strains of the results of these two hierarchical clustering algorithms, while the other two clusters are different, and the strains of them are, respectively, AY.29 and BF.28 against BA.2 and BA.5.1.3 strains. However, there is only two clusters consistent to that of the above two algorithms when utilizing Ward-Jaccard algorithm, and they are represented by the strains of A.17 and BE.1.1.1, respectively. The other four clusters of the Ward-Jaccard algorithm are different to that of the above two algorithms’, and the strains of them are A, BA.5.2.34, BA.2.10 and BQ.1.14 strains, respectively.

From the results of Method (2), it can be inferred that no matter which clustering algorithm is utilized among three ones, the strains of A.17 and BE.1.1.1 exhibit distinct characteristics compared to other clusters’. However, A.17 emerged only about one month before it was discovered and was associated with only 11 cases, suggesting that it was rapidly eliminated and might not have gained an advantage in competition with other strains based on its sequence composition. A.17 had three mutations (L53F, Q116H, and Q185H) in the ORF3a region.

Regarding the results of Ward-Euclidean algorithm combining Method (2) presented in Table 1, that is, with respect to the clustering of Fig. 3a, apart from A.17, two strains belonged to the Delta variant, namely AY.29 and AY.126. As they belong to the same variant, their sequence features are mostly similar, with AY.29 having an additional mutation (V1750A) in the ORF1a region compared to AY.126. AY.126 exhibits mutations (K2557R, I850L, and K16T) in the ORF1b, S protein, and ORF3a regions, respectively. AY.29 appeared later than AY.126 but accumulated more cases, and both of them disappeared around the same time. The strains representing remaining three clusters are BE.1.1.1, BA.5.5, and BF.28, belonging to the Omicron variant. They shared similar sequence features, with BF.28 having an additional mutation (Q44L) in the ORF1a region, leading to its designation as BF.28. The BE.1.1.1 strain had additional mutations (L3829F, M1156I, K444T, and E136D) in the ORF1a, ORF1b, S protein, and N protein regions, respectively. The K444T mutation is considered a major antigen targeted SARS-CoV-2 by vaccines and therapeutic monoclonal antibodies^28^. The BF.28 emerged in July of 2020, while BE.1.1.1 and BA.5.5 appeared in the early of 2022, indicating a later emergence but stronger transmissibility, aligning with viral evolution trends. The detail information of these strains can refer to the Table S1 in Supplementary materials.

Compared to the results of Ward-Euclidean algorithm based on Method (2) to select the representative samples, the results of the Average-Euclidean algorithm combining with Method (2) includes two distinct strains, BA.2 and BA.5.1.3, both belonging to the Omicron variant. The BA.2 strain exhibits unique mutations in ORF1a, S protein, and ORF6, specifically L3201F, Q493R, and D61L. Notably, the Q493R mutation has the potential to facilitate interspecies transmission and requires vigilance^29^. On the other hand, the BA.5.1.3 strain carries distinct mutations, D3N and L37F, in the M protein and ORF10, respectively. Additionally, it presents four additional mutations in the S protein compared to BA.2, namely DEL69/70, V289I, L452R, and F486V. The L452R and F486V mutations in particular contribute to the enhanced immune evasion capability in the BA.5 variant. Genetic sequencing analysis reveals minimal differences between BA.5.1.3 and BA.5, with the former possessing an extra mutation in the S protein. However, this additional mutation does not impact their pathogenicity, immune evasion, or transmissibility, suggesting similar infectivity between the two strains^30,31^. The L452R mutation, situated in the receptor-binding domain (RBD), increases the virus’s infectivity and has been associated with evading neutralizing antibodies^27^.

It should be noted that BA.2 strain appeared before the BA.5.1.3 strain in terms of timeline and continues being present, with its spread being considerably more widespread than that of BA.5.1.3 strain. The timeline of each strain can be noticed in Table S1 in Supplementary materials.

The strains of the clustering of Ward-Jaccard algorithm based on Method (2), both BA.5.2.34 and BQ.1.14 exhibit the L452R and F486V mutations mentioned earlier, while BA.2.10 does not possess these mutations. Additionally, in comparison to BA.2.10 strain, the BA.5.2.34 and BQ.1.14 strains show closer similarities, indicating a closer evolutionary relationship between BA.5.2.34 and BQ.1.14 strains. Regarding their timeline shown in Table S1 in Supplementary materials, the BA.2.10 strain emerged in the April of 2020, while the BA.5.2.34 and BQ.1.14 strains appeared only in the early of 2022. These three SARS-CoV-2 strains are still diagnosed in 2023 as well.

The results based on the representative samples selected by utilizing Method (3) and shown in Table 1 demonstrate that algorithms Ward-Euclidean and Average-Euclidean yield three identical clusters with strains of BA.5.6.4, BA.5.3, and BF.28. Conversely, three different clusters are with strains of A.17, BE.1.1.1, and CH.1.1 compared to BA.5.5, BF.3.1, and BQ.1.1.18, respectively. Furthermore, when utilizing Ward-Jaccard algorithm, only the strain of BA.5.6.4 aligns with the results of Ward-Euclidean and Average-Euclidean algorithms, while the remaining five variants presenting the other five clusters are identified as BA.2.2.1, AY.127, AY.29, BQ.1.14, and BQ.1.1.19 strains.

In the results of Ward-Euclidea clustering algorithm utilizing Method (3) to select representative samples, the strains of BA.5.6.4, BA.5.3, BF.28, BE.1.1.1, and CH.1.1 are classified as Omicron variants. The CH.1.1 strain represents the sixth-generation sub-branch of the Omicron variant of BA.2.75^32^. Compared to the preceding four variants, CH.1.1 exhibits a greater number of mutations in the S protein, including R346T, F486S, and K444T, which are major antigens targeted by vaccines and therapeutic monoclonal antibodies against SARS-CoV-2^32^.

In the results of Average-Euclidean algorithm utilizing Method (3) to select representative samples, all six variants of its clusters are classified as Omicron strains. Among them, BQ.1.1.18 exhibits significant differences in mutations in ORF1b and the S protein compared to the other five variants from the rest five clusters, while the remaining regions show relatively close similarity.

In the results of Ward-Jaccard clustering algorithm combining Method (3) for selecting representative samples, The strains of AY.127 and AY.29 are categorized as Delta variants, while the other four variants of the rest four clusters belong to the Omicron lineage. Notably, BA.2.2.1 distinguishes itself from other strains by possessing three acquired mutations: S:I1221T, ORF1a:T1543I, ORF1a:T4087I. Additionally, ORF8:I76V is also a characteristic feature of BA.2.2.1 strain^33^. On the other hand, BQ.1.14 and BQ.1.1.19 strains exhibit high similarity, with BQ.1.1.19 showing only three additional mutationsin ORF1b, the S protein, and the N protein: N1191S, R346T, and Q380H, compared to BQ.1.14 strain.

The results based on Method (4) demonstrate that when using Average-Euclidean algorithm, the results are identical to those obtained by the same clustering algorithm based on Method (3). However, when using Ward-Euclidean clustering algorithm, the variant of a cluster is different from that of the corresponding cluster obtained through Method (3). Similarly, when using Ward-Jaccard algorithm, three variants of three clusters are consistent with the results obtained by same algorithm based on Method (3), namely BA.5.6.4, BA.2.2.1, and AY.127 strains, while the other three clusters possess three different variants of AY.4, BA.2, and B.1.1.317 strains. This suggests that Methods (3) and (4), which involve selecting representative samples, yield comparable results to a certain extent.

Among the strains of Ward-Jaccard algorithm based on Method (4), the AY.127 and AY.4 strains are classified as Delta variants, while the other four variants belong to the Omicron lineage. Notably, B.1.1.317 exhibits relatively fewer mutations, with only a few mutations present in ORF1b, the S protein, ORF8, and the N protein.

In conclusion, the strains observed in China primarily belong to the Delta and Omicron lineages. The results from Methods (3) and (4) show similarities, indicating that these two representative sample selection methods proposed in this paper lead to the comparable outcomes. On the other hand, Methods (1) and (2) yield more significant differences in their results.

Among the results of the aforementioned methods proposed in this paper, several strains have the highest frequency of occurrence, including BF.28, BE.1.1.1, BA.5.3, and BA.5.6.4. This suggests that these four strains likely possess distinct characteristics not present in other strains, making them potential major sources of future viral evolution. Additionally, attention should be given to strains such as BA.2.75, CH.1.1, BA.2, BA.5.1.3, BF.7, and B.1.1.214, which exhibit enhanced abilities in immune evasion, transmissibility, and pathogenicity. Monitoring their future evolutionary trends is of utmost importance.

The utilization of the combined approaches allows us to gain a comprehensive understanding of the relationships among the COVID-19 virus strains, shedding light on their origins, evolution, and transmission patterns. This is crucial for disease surveillance, vaccine development, and disease control, while providing valuable insights for informing public health strategies and implementing effective measures.

## Conclusion

This article primarily focuses on conducting hierarchical clustering analysis to all of the SARS-CoV-2 strains detected in China before January 7, 2023, so as to discover the evolutionary relationship between the strains of SARS-CoV-2 coronavirus. Four representative sample selection methods were proposed in this paper to select samples from different types of strains of SARS-CoV-2 coronavirus for the subsequent hierarchical clustering analysis. Three hierarchical clustering algorithms named Ward-Euclidean, Ward-Jaccard, and Average-Euclidean were proposed to investigate the evolutionary relationship between the SARS-CoV-2 coronavirus strains. Moreover, the famous *k*-mer algorithm was utilized to encode the genetic sequences of the SARS-CoV-2 strains into numerical data firstly for the hierarchical clustering analysis.

The hierarchical clustering analysis detected six clusters of the SARS-CoV-2 strains identified in China. Extensive experimental results analyses and reasonable inferences found that BF.28, BE.1.1.1, BA.5.3, and BA.5.6.4 strains are the popular and potential major sources of future viral evolution strains. Additionally, it was found that BA.2.75, CH.1.1, BA.2, BA.5.1.3, BF.7, and B.1.1.214 strains exhibit enhanced abilities in immune evasion, transmissibility, and pathogenicity, which indicates that further insightful study and investigation from molecular level need paying attention to these strains. Some latest studies^34–36^ have also provided the specific proof to these findings. Monitoring should also be given to the future evolutionary trends of these SARS-CoV-2 strains. The public health interventions need paying much attention to these strains in the future. This study investigated the relationship among the SARS-CoV-2 strains identified in China. Although the dataset utlized in this study may not capture the global diversity of SARS-CoV-2 strains, it will provide valuable insights into the relationships of the available SARS-CoV-2 strains and their evolutionary trends, and will also provide some assistance and valuable insights for future research on the SARS-CoV-2 virus, minimizing the impact of future SARS-CoV-2 variants on humanity and countries. However, we only conduct hierarchical clustering analysis to the SARS-CoV-2 strains detected in China before January 7, 2023. Although the pandemic is not serious after January, 2023, the coronavirus still exists in China, particularly in the past May and summer holiday. Therefore, the relationship of the newest and the having existing strains of SARS-CoV-2 variants need further studying.

### Supplementary Information

Supplementary Information.

## Data Availability

The dataset utilized in this study was obtained from the global initiative on sharing all influenza data (GISAID)(https://gisaid.org/). This dataset comprises all available genomic sequences of SARS-CoV-2 collected in China till January 7, 2023.
