# Supplementary material for: Clustering analysis for the evolutionary relationships of SARS-CoV-2 strains
Source: Sci Rep. 2024 Mar 18;14:6428. doi: 10.1038/s41598-024-57001-5 (PMC10948388; doi:10.1038/s41598-024-57001-5)
Supplement: Supplementary file 1 — Supplementary Information. [file 41598_2024_57001_MOESM1_ESM.docx]

Clustering analysis of the evolutionary relationships of SARS-CoV-2 strains

Xiangzhong Chen^1,+^, Mingzhao Wang^1,+^, Xinglin Liu^1^, Wenjie Zhang^1^, Huan Yan^1^, Xiang Lan^1^, Yandi Xu^2^, Sanyi Tang ^3,#^, Juanying Xie^1,#^

^1^ School of Computer Science, Xian, 710119, China

^2^ College of Life Sciences, Shaanxi Normal University, Xi’an, 710119, China

^3^ School of Mathematics and Statistics, Xian, 710119, China

+ Co-first author, # Co-corresponding author

E-mail：[xiejuany@snnu.edu.cn, sytang@snnu.edu.cn](mailto:xiejuany@snnu.edu.cn,%20sytang@snnu.edu.cn,%20xushengquan@snnu.edu.cn)

**Table S1：** The earliest occurrence time and other information of all strains in the clustering results in Table 1 **(The deadline is at 10:20, March, 10, 2023)**

| Strain type | Earliest occurrence time | Last time of discovery | total number | other information |
| --- | --- | --- | --- | --- |
| BQ.1.1.19 | 2022-09-27 | 2023-02-15 | 453 | Alias of B.1.1.529.5.3.1.1.1.1.1.1.19, England, N:Q380H |
| BN.1.5 | 2022-08-15 | 2023-02-28 | 3208 | Alias of B.1.1.529.2.75.5.1.5, ORF1a:S2103F |
| BA.5.6.4 | 2022-07-03 | 2023-01-24 | 143 | Alias of B.1.1.529.5.6.4, found in USA and Europe |
| BF.3.1 | 2022-06-01 | 2022-09-30 | 377 | Alias of B.1.1.529.5.2.1.3.1, mainly found in India, Singapore, and USA |
| BF.27 | 2022-02-18 | 2023-01-25 | 7245 | Alias of B.1.1.529.5.2.1.27, Singapore lineage, defined by ORF1a:A2784V |
| BE.1.1.1 | 2022-02-06 | 2023-02-23 | 2545 | Alias of B.1.1.529.5.3.1.1.1.1, found globally |
| BA.5.5 | 2022-01-10 | 2023-02-22 | 58193 | Alias of B.1.1.529.5.5, mainly found in USA |
| CH.1.1 | 2022-01-09 | 2023-03-08 | 17697 | Alias of B.1.1.529.2.75.3.4.1.1.1.1, defined by S:L452R |
| BA.5.3 | 2022-01-05 | 2023-02-13 | 4331 | Alias of B.1.1.529.5.3, mainly found in Germany and South Africa |
| BQ.1.14 | 2022-01-05 | 2023-03-01 | 5212 | Alias of B.1.1.529.5.3.1.1.1.1.1.14, international lineage, 18570A |
| BA.2.2.1 | 2022-01-03 | 2022-08-02 | 1079 | —— |
| BA.5.2.34 | 2022-01-02 | 2023-02-24 | 4063 | Alias of B.1.1.529.5.2.34, mainly found in Israel and USA |
| BF.7 | 2022-01-02 | 2023-02-27 | 60388 | Alias of B.1.1.529.5.2.1.7, mainly found in Belgium, England and Denmark |
| BA.2.75 | 2021-12-31 | 2023-02-20 | 6138 | Alias of B.1.1.529.2.75, mainly found in India |
| BQ.1.1.18 | 2021-12-28 | 2023-03-08 | 7358 | Alias of B.1.1.529.5.3.1.1.1.1.1.1.18, France, C6541T |
| BA.1.13 | 2021-11-16 | 2022-10-04 | 4839 | Alias of B.1.1.529.1.13, Indonesia lineage |
| BA.5.1.3 | 2021-09-17 | 2023-02-13 | 11033 | Alias of B.1.1.529.5.1.3, mainly found in Germany Portugal and Spain |
| AY.30 | 2021-03-27 | 2022-05-02 | 3868 | —— |
| AY.57 | 2021-01-30 | 2022-06-20 | 2895 | —— |
| AY.29 | 2021-01-28 | 2022-05-13 | 86808 | Evolved from AY. 4 |
| AY.127 | 2021-01-09 | 2022-11-25 | 28251 | Alias of B.1.617.2.127, lineage in India and other countries |
| AY.126 | 2020-10-14 | 2022-05-09 | 43026 | —— |
| BF.28 | 2020-07-22 | 2023-02-09 | 9710 | Alias of B.1.1.529.5.2.1.28, Singapore lineage, defined by ORF1a:Q44L |
| BF.5 | 2020-07-21 | 2023-02-27 | 83419 | Alias of B.1.1.529.5.2.1.5, Israel lineage |
| BA.5 | 2020-07-04 | 2023-02-23 | 24728 | Alias of B.1.1.529.5 |
| BA.5.2 | 2020-07-01 | 2023-02-28 | 290,508 | Alias of B.1.1.529.5.2, mainly found in South Africa, England and USA |
| AY.4 | 2020-05-11 | 2022-09-28 | 858，395 | Alias of B.1.617.2.4, UK lineage |
| BA.2.10 | 2020-04-03 | 2023-02-15 | 67051 | Alias of B.1.1.529.2.10 |
| BA.2 | 2020-03-28 | 2023-03-01 | 1220,995 | Alias of B.1.1.529.2 |
| B.1.1.317 | 2020-03-19 | 2022-06-06 | 2808 | Not considered a noteworthy variant |
| A.17 | 2020-03-02 | 2020-03-28 | 11 | —— |
| B.1.1.214 | 2020-02-22 | 2021-07-29 | 19,687 | —— |
| A | 2019-12-30 | 2023-02-16 | 3108 | One of the two original haplotypes of the pandemic (A and B). Many sequences originating from China and many global exports; including to South East Asia Japan South Korea Australia the USA and Europe represented in this lineage |
